# Prediction of Overall Survival Among Female Patients With Breast Cancer Using a Prognostic Signature Based on 8 DNA Repair–Related Genes

**DOI:** 10.1001/jamanetworkopen.2020.14622

**Published:** 2020-10-05

**Authors:** Dai Zhang, Si Yang, Yiche Li, Jia Yao, Jian Ruan, Yi Zheng, Yujiao Deng, Na Li, Bajin Wei, Ying Wu, Zhen Zhai, Jun Lyu, Zhijun Dai

**Affiliations:** 1 Department of Breast Surgery, The First Affiliated Hospital, College of Medicine, Zhejiang University, Hangzhou, China; 2 Department of Oncology, The Second Affiliated Hospital of Xi’an Jiaotong University, Xi’an, China; 3 Breast Center Department, The Fourth Hospital of Hebei Medical University, Hebei Medical University, Shijiazhuang, China; 4 Department of Medical Oncology, The First Affiliated Hospital, College of Medicine, Zhejiang University, Hangzhou, China; 5 Department of Clinical Research, The First Affiliated Hospital of Jinan University, Guangzhou, China

## Abstract

**Importance:**

Breast cancer (BC), a common malignant tumor, ranks first among cancers in terms of morbidity and mortality among female patients. Currently, identifying effective prognostic models has a significant association with the prediction of the overall survival of patients with BC and guidance of clinicians in early diagnosis and treatment.

**Objectives:**

To identify a potential DNA repair–related prognostic signature through a comprehensive evaluation and to further improve the accuracy of prediction of the overall survival of patients with BC.

**Design, Setting, and Participants:**

In this prognostic study, conducted from October 9, 2019, to February 3, 2020, the gene expression profiles and clinical data of patients with BC were collected from The Cancer Genome Atlas database. This study consisted of a training set from The Cancer Genome Atlas database and 2 validation cohorts from the Gene Expression Omnibus, which included 1096 patients with BC. A prognostic signature based on 8 DNA repair–related genes (DRGs) was developed to predict overall survival among female patients with BC.

**Main Outcomes and Measures:**

Primary screening prognostic biomarkers were analyzed using univariate Cox proportional hazards regression analysis and the least absolute shrinkage and selection operator Cox proportional hazards regression. A risk model was completely established through multivariate Cox proportional hazards regression analysis. Finally, a prognostic nomogram, combining the DRG signature and clinical characteristics of patients, was constructed. To examine the potential mechanisms of the DRGs, Gene Ontology and Kyoto Encyclopedia of Genes and Genomes enrichment analyses were performed.

**Results:**

In this prognostic study based on samples from 1096 women with BC (mean [SD] age, 59.6 [13.1] years), 8 DRGs (*MDC1*, *RPA3*, *MED17*, *DDB2*, *SFPQ*, *XRCC4*, *CYP19A1*, and *PARP3*) were identified as prognostic biomarkers. The time-dependent receiver operating characteristic curve analysis suggested that the 8-gene signature had a good predictive accuracy. In the training cohort, the areas under the curve were 0.708 for 3-year survival and 0.704 for 5-year survival. In the validation cohort, the areas under the curve were 0.717 for 3-year survival and 0.772 for 5-year survival in the GSE9893 data set and 0.691 for 3-year survival and 0.718 for 5-year survival in the GSE42568 data set. This DRG signature mainly involved some regulation pathways of vascular endothelial cell proliferation.

**Conclusions and Relevance:**

In this study, a prognostic signature using 8 DRGs was developed that successfully predicted overall survival among female patients with BC. This risk model provides new clinical evidence for the diagnostic accuracy and targeted treatment of BC.

## Key Points

**Question:**

Can a better prognosis model based on DNA repair–related genes be developed for the comprehensive evaluation of breast cancer?

**Findings:**

In this prognostic study based on samples from 1096 women, a novel signature for breast cancer constructed using 8 DNA repair–related genes showed satisfactory performance for predicting survival in the training cohort from The Cancer Genome Atlas and the validation cohort from the Gene Expression Omnibus database. The DNA repair–related gene signature was an independent predictor of survival after adjustment for clinical characteristics.

**Meaning:**

A prognostic signature based on 8 DNA repair–related genes predicted overall survival among female patients with breast cancer, thereby providing a new modality for the accurate diagnosis and targeted treatment of breast cancer.

## Teaser

This prognostic study evaluates a potential DNA repair–related prognostic signature to improve the accuracy of prediction of the overall survival of patients with breast cancer.

## Introduction

Global Cancer Statistics 2018 estimated that 18.1 million new cases of cancer and 9.6 million cancer-related deaths occurred globally in 2018.^1^ In comparison with the 1 345 680 cancer-related deaths that occurred in the United States in 2014, the total number of cancer-related deaths in this country in 2019 has been estimated to increase by approximately 4.8% (1 409 700 cancer-related deaths, including 787 800 men and 621 900 women) based on the latest cancer prediction data.^2^ In terms of incidence and mortality, the global burden of female breast cancer (BC) is large and is still increasing in several countries.^3^ In addition, BC is still deemed to be the most common cause of cancer-related death among individuals with cancer and among women worldwide.^1^ The significant improvements in the quality of life and the life expectancy of patients with BC may be associated with the progress in BC treatment. Nevertheless, the improvement in the overall clinical outcome of patients is still crucial.^4^ Moreover, the mortality of BC remains a global challenge.^5^ Therefore, it is necessary to identify effective prognostic models to assess the overall survival (OS) of patients with BC and provide guidance for clinicians in early diagnosis and treatment.

With the advancement of genome-sequencing technologies, accumulating evidence has shown that gene signatures have the potential for predicting BC prognosis. For example, a positron emission tomography signature of 242 genes that reflects high glucose uptake is a novel, reliable, independent prognostic factor for BC.^6^ Another prognostic signature of BC based on 8 long noncoding RNAs (TFAP2A-AS1, CHRM3-AS2, MIAT, DIAPH2-AS1, NIFK-AS1, LINC00472, MEF2C-AS1, and WEE2-AS1) showed a moderate predictive ability for 5-year OS (area under the curve [AUC] for training set, 0.65).^7^ Seven DNA methylation sites have a good prognostic performance for OS (AUC = 0.74).^8^ An 8-long noncoding RNA signature (AC007731.1, AL513123.1, C10orf126, WT1-AS, ADAMTS9-AS1, SRGAP3-AS2, TLR8-AS1, and HOTAIR) performed with insufficient accuracy as a potential indicator for predicting survival (AUC = 0.692).^9^ Some of the existing prognostic models for BC lack excellent accuracy and a comprehensive assessment. Recently, molecular biomarkers for the diagnosis or prognosis of BC, including DNA repair–related genes (DRGs), have gained attention in the field of oncology.^10,11,12,13^ Genome stability (to prevent cell death or neoplastic transformation) and DNA damage response involve the activation of numerous cellular activities that repair DNA lesions and maintain genomic integrity, which are critical in preventing tumorigenesis.^14^ DNA damage repair is found to be associated with BC resistance, which, in turn, is associated with the prognosis of patients. Studies on targeted therapy of the DNA damage response pathway have made new progress.^15^ However, there is no currently accurate prediction signature for DRGs, to our knowledge; therefore, the present study aimed to develop a better prognosis model based on DRGs via a comprehensive evaluation.

Clinical models based on multiple independent prognostic factors achieve higher prognostic prediction accuracy for patients with BC compared with models that use a single gene or clinical biomarker. Currently, data for large BC sample cohorts, including clinical characteristics and corresponding transcriptome profiles, can be obtained from The Cancer Genome Atlas (TCGA) database^16^; in addition to these data, a large series of publicly available gene expression data sets for validation were collected from the Gene Expression Omnibus database for the present study. In this study, we constructed a DRG-based prognostic model to predict 3- and 5-year OS. Moreover, our prognostic model integrates a newly identified 8-DRG signature and other independent clinical risk features, which have been evaluated and validated in patients with BC. A functional enrichment analysis was performed to identify probable hub pathways in which the 8 DRGs might be involved. Finally, we tested the ability of the newly constructed 8-DRG signature to predict prognosis and OS among patients with BC.

## Methods

### Data Sourcing and Differential Expression Analysis

In this prognostic study, conducted from October 9, 2019, to February 3, 2020, the transcriptome RNA-sequencing data of BC samples were obtained from the TCGA data portal, and the corresponding clinical information was also obtained. All of the data are publicly available from the US National Cancer Institute.^17^ The exclusion criteria were as follows: BC in a male patient, confirmed non-BC pathologic diagnosis, and patients with BC with incomplete information regarding clinical characteristics (age, sex, survival time, survival status, pathologic stage, estrogen receptor [ER; positive or negative] status, progesterone receptor [PR; positive or negative] status, ERBB2 [positive or negative] status, and TNM stage). In total, 707 BC tissue samples and 112 samples from healthy controls from the TCGA data set were included in our analysis. A comprehensive list of DRGs was obtained online from the UALCAN website^18,19^ and from previous studies.^13,20^ The expression profiles of DRGs were extracted from transcriptome RNA sequencing data of the BC tissue samples. In addition, the differentially expressed genes (DEGs) were evaluated using the limma R package (R Foundation for Statistical Computing).^21^ The genes with an absolute log_2_-fold change of more than 1 and an adjusted *P* < .05 were considered for subsequent analysis. Heat maps were created using the pheatmap package of the R, version 3.5.2 software. This study was conducted in accordance with the Transparent Reporting of a Multivariable Prediction Model for Individual Prognosis or Diagnosis (TRIPOD) guidelines.^22^ Detailed descriptions of the methods are in eTable 1 in the Supplement. In addition, this study was approved by the institutional review board of the First Affiliated Hospital of Zhejiang University, Zhejiang Province, Hangzhou, China. Informed consent was waived because the study does not involve specimen collection and is a secondary analysis of public data.

### Statistical Analysis

#### Construction and Evaluation of the 8-DRG Prediction Model

We performed a univariate Cox proportional hazards regression analysis to assess the association between the expression levels of the DRGs and patient OS, setting an adjusted *P* < .05 as the cutoff for statistical significance. Then, the least absolute shrinkage and selection operator (LASSO) method^23^ was used for further screening of prognostic DRGs. Finally, a multivariate Cox proportional hazards regression analysis was performed to construct a DRG-derived prognostic model based on the Akaike information criterion. The data sets (GSE9893 and GSE42568) for validation of the robustness of the 8-DRG prognostic model were downloaded from the Gene Expression Omnibus database.^24^ A time-dependent receiver operating characteristic (ROC) curve was created to investigate whether the built model could effectively predict survival of patients with BC using the timeROC R package. The survival rates of the patients in the high-risk and low-risk groups were estimated using the Kaplan-Meier survival curve and the survival R package.

#### 8-DRG Signature as an Independent Prognostic Factor

To verify that the 8-DRG signature was independent of other clinical characteristics, univariate and multivariate Cox proportional hazards regression analyses were performed. First, a univariate Cox proportional hazards regression analysis was used to identify the clinical features associated with the OS of patients with BC. Then, a multivariate Cox proportional hazards regression analysis was used to evaluate whether the 8-DRG signature could be an independent indicator of OS after adjusting for other traits. Accordingly, a nomogram was established on the basis of the results of the multivariate Cox proportional hazards regression analysis to obtain an individual prediction of OS.

#### Functional Enrichment Analysis

To analyze the Gene Ontology and Kyoto Encyclopedia of Genes and Genomes terms of the DRG-related signature, a gene set enrichment analysis was performed between the high-risk and low-risk groups using the clusterProfiler R software package.^25^ The criterion for screening out significant terms was *P* < .05.

## Results

### DEGs in Patients With BC

**Figure 1.**
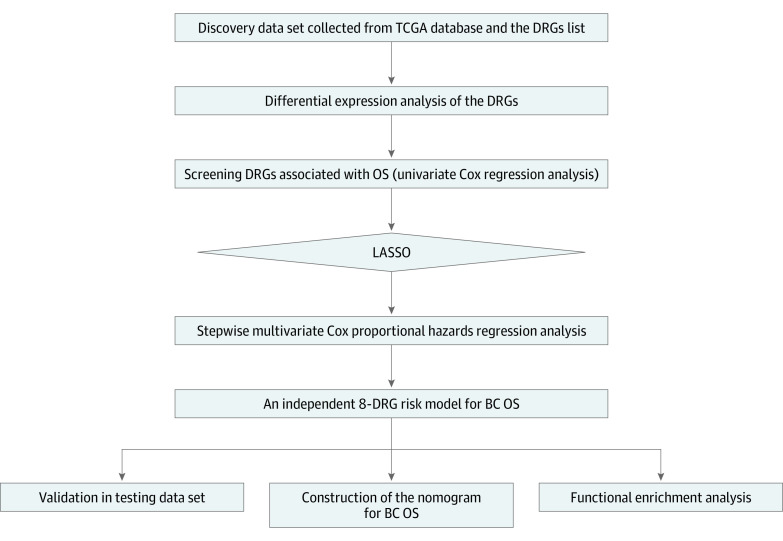
Flowchart of the Research Design BC indicates breast cancer; DRGs, DNA repair–related genes; LASSO, least absolute shrinkage and selection operator; OS, overall survival; and TCGA, The Cancer Genome Atlas.

In accordance with the defined criteria, RNA sequencing expression profiles and clinical information for 707 BC tissues and for tissues from 112 healthy controls (mean [SD] age, 58.0 [13.3] years; mean [SD] follow-up time, 3.4 [3.3] years) were downloaded from the TCGA data portal. The integrated clinical data are provided in eTable 2 in the Supplement, and the list of the 513 genes is provided in eTable 3 in the Supplement. Figure 1 shows the flowchart of the study procedure. A total of 496 DEGs (343 upregulated and 153 downregulated) were identified from the set of 513 DRGs (eTable 4 in the Supplement). The expression heat map of the DEGs is presented in eFigure 1 in the Supplement.

### Construction and Evaluation of the Gene Prediction Model

**Figure 2.**
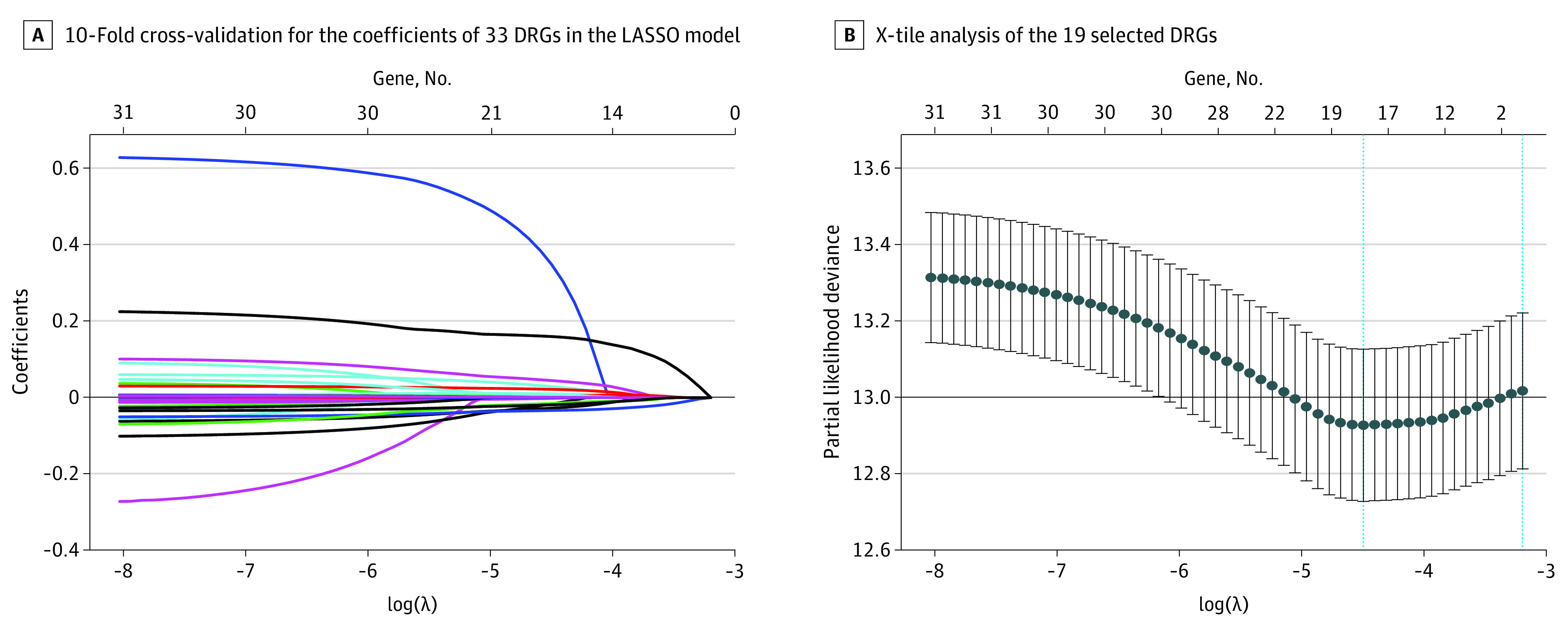
Selection of DNA Repair–Related Genes (DRGs) Using the Least Absolute Shrinkage and Selection Operator (LASSO) Model Error bars indicate 95% CIs.

**Figure 3.**
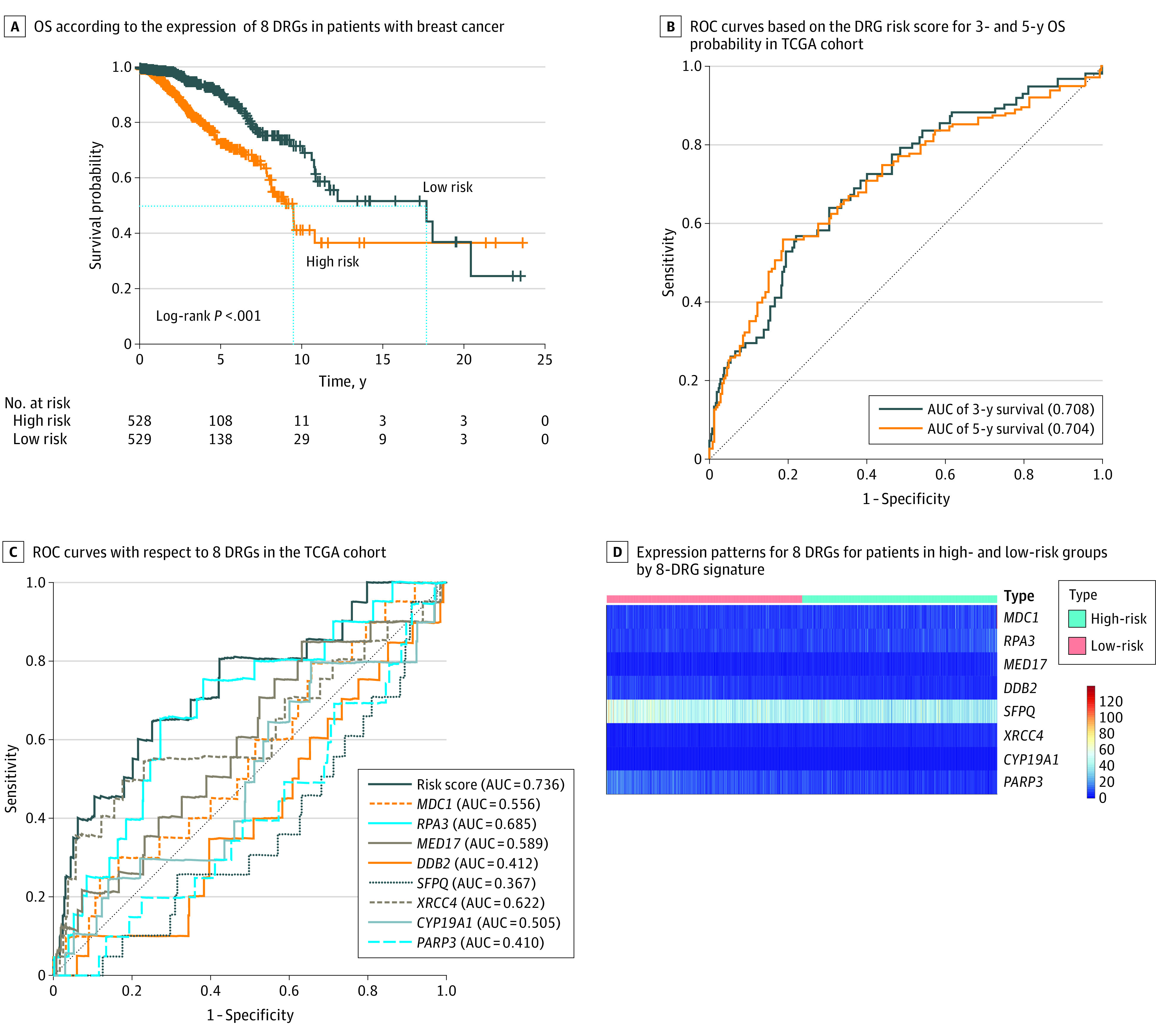
Prognostic Role of the Signature Using 8 DNA Repair–Related Genes (DRGs) in the Training Set A, Kaplan-Meier plots of overall survival (OS) according to the expression of 8 key DRGs in patients with breast cancer. B, Receiver operating characteristic (ROC) curves based on the DRG risk score for 3- and 5-year OS probability in The Cancer Genome Atlas (TCGA) cohort. C, ROC curves with respect to 8 key DRGs in the TCGA cohort. D, Expression patterns for DRGs for patients in high- and low-risk groups by the 8-DRG signature. AUC indicates area under the curve.

**Figure 4.**
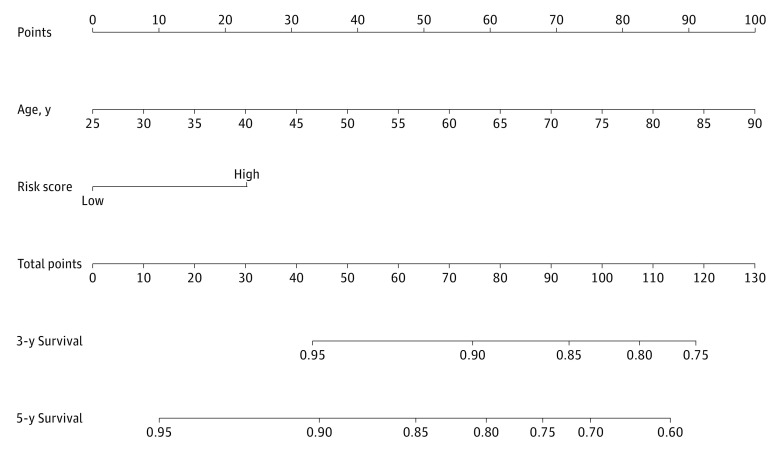
Nomogram for Predicting Probabilities of Overall Survival in Patients With Breast Cancer

A total of 33 DEGs with potential prognostic value were identified through the univariate Cox proportional hazards regression analysis (eTable 5 in the Supplement), with 19 remaining after filtration using LASSO (Figure 2). Finally, 8 DRGs (*MDC1* [OMIM 607593], *RPA3* [OMIM 179837], *MED17* [OMIM 603810], *DDB2* [OMIM 600811], *SFPQ* [OMIM 605199], *XRCC4* [OMIM 194363], *CYP19A1* [OMIM 107910], and *PARP3* [OMIM 607726]) were selected to construct a prediction model using multivariate Cox proportional hazards regression analysis (eFigure 2 in the Supplement). The total risk score was imputed as follows: (0.029083098 × *MDC1* expression level) + (0.054759912 × *RPA3* expression level) + (0.085847823 × *MED17* expression level) + (−0.06445 × *DDB2* expression level) + (−0.02875298 × *SFPQ* expression level) + (0.234473864 × *XRCC4* expression level) + (0.567390823 × *CYP19A1* expression level) + (−0.065799614 × *PARP3* expression level). The patients with high-risk scores had a poor prognosis (Figure 3A). The AUCs of the time-dependent ROC curve were 0.708 for 3-year survival and 0.704 for 5-year survival (Figure 3B). The AUCs of the time-dependent ROC curve for the single genes were 0.556 for *MDC1*, 0.685 for *RPA3*, 0.589 for *MED17*, 0.412 for *DDB2*, 0.367 for *SFPQ*, 0.622 for *XRCC4*, 0.505 for *CYP19A1*, and 0.410 for *PARP3* (Figure 3C). The expression of 8 DRGs for patients with BC in the total set are displayed in Figure 3D. Nomograms of the 3-year and 5-year OS in the cohort are presented in Figure 4.

### External Validation Set and Performance

To validate the prognostic predictive ability of the 8-DRG signature, the GSE9893 data set reported in 2008, with 155 records of patients with BC (mean [SD] age, 67.3 [10.2] years; mean [SD] follow-up time, 6.0 [2.4] years), and the GSE42568 data set reported in 2006, with 122 records of patients with BC (mean [SD] age, 58.0 [11.7] years; the mean [SD] follow-up time, 5.2 [2.4] years), were chosen. The whole validation group was divided into high-risk and low-risk groups in the discovery data set. In GSE9893, the AUC was 0.717 for 3-year OS and 0.718 for 5-year OS. In GSE42568, the AUC was 0.691 for 3-year OS and 0.718 for 5-year OS (eFigure 3 A and B in the Supplement). The survival analysis revealed a good performance of the risk model for stratifying high-risk and low-risk patients (eFigure 3 C and D in the Supplement).

### Risk Score of the 8-DRG Signature as an Independent Indicator for Predicting BC Prognosis

To develop a composite predictor of OS for patients with BC, we combined the 8-DRG signature and clinicopathologic characteristics for the screening of the independent predictive factors of OS (Table). The univariate Cox proportional hazards regression analysis result indicated that high risk was significantly associated with shorter survival in the TCGA cohort (hazard ratio, 2.01; 95% CI, 1.21-3.32; *P* = .007). After multivariate adjustment using these factors, the risk score remained an independent prognostic factor (hazard ratio, 1.39; 95% CI, 1.08-2.40; *P* = .02) in this set. The calibration curves for the probability of survival at 3 and 5 years showed a good agreement between the prediction based on the nomogram and the actual observations (eFigure 4 A and B in the Supplement).

**Table.** Risk Score Generated From the 8-DRG Signature as an Independent Indicator According to Cox Proportional Hazards Regression Model

Variable	Univariate analysis		Multivariate analysis	
	HR (95% CI)	*P* value	HR (95% CI)	*P* value
Age, y (>60 or ≤60)	1.03 (1.01-1.04)	.008	1.89 (1.13-3.17)	.02
Pathologic stage (I, II, III, or IV)	1.74 (1.28-2.37)	<.001	1.64 (0.80-3.36)	.18
ER (negative or positive)	0.67 (0.40-1.21)	.13	NA	NA
PR (negative or positive)	0.56 (0.34-0.90)	.02	0.43 (0.20-1.03)	.06
ERBB2 (negative or positive)	0.93 (0.44-1.94)	.84	NA	NA
Pathologic T (T1 and T2 or T3 and T4)	1.20 (0.91-1.60)	.20	NA	NA
Pathologic N (N0 and N1 or N2 and N3)	1.59 (1.22-2.08)	<.001	1.22 (0.77-1.93)	.39
Metastasis (M0 or M1)	3.73 (1.70-8.19)	.001	1.06 (0.29-2.99)	.91
8-DRG risk scores (high or low)	2.01 (1.21-3.32)	.007	1.39 (1.08-2.40)	.02

Abbreviations: DRG, DNA repair–related gene; ER, estrogen receptor; HR, hazard ratio; NA, not applicable; PR, progesterone receptor.

### Functional Enrichment Analysis of the DRGs

The patients with BC identified from the TCGA database were divided into high-risk and low-risk subgroups according to the median of all patients’ risk scores. A gene set enrichment analysis was performed to further investigate the potential biological processes and examine the associated mechanisms of these 2 groups. Gene Ontology analyses revealed that some angiogenesis regulation pathways (negative regulation of vascular endothelial cell proliferation, regulation of artery morphogenesis, vascular endothelial cell proliferation, and vascular endothelial cell proliferation) were the main enriched pathways in the high-risk group (eFigure 5 A and B in the Supplement). In addition, the Kyoto Encyclopedia of Genes and Genomes pathway enrichment analysis in the high-risk and low-risk groups are shown in eTable 6 and eFigure 6A and B in the Supplement. The 3 cancer-related pathways (ie, the hedgehog signaling, retinoic acid-inducible gene 1–like receptor signaling, and cytosolic DNA-sensing pathways) were found to be enriched in the high-risk group.

## Discussion

The incidence of BC ranks first in the US and global rankings. Mortality due to BC ranks second in the US and first worldwide. Breast cancer is a significant cause of morbidity and premature mortality among women globally.^4,26^ More efforts are needed to achieve a good prognosis for BC, which is still considered a challenge. Clinical management places emphasis on the importance of early and effective detection and prediction of prognosis, with the aim of achieving precise individualized treatment. The application of prognostic models is useful for guiding clinical decisions and is essential for precision medicine. However, for reasons such as insufficient sample size and lack of verification in other external cohorts, the prognosis and prediction capabilities of the current BC prognostic models are not satisfactory.^8,9,27^

Studies on DNA repair pathways and DRGs have found some new results. Inactivation of DRGs can disrupt genome integrity, which can increase the risk of the accumulation of gene mutations associated with cancer development.^28^ Some reports suggest that the DNA repair process is involved in the intrinsic response of the body to chemotherapeutic agents and has been shown to be associated with the mechanisms of resistance acquired during treatment.^28,29^ Some DRG prognostic biomarkers of BC have been identified so far.^13^ Hence, our study aimed to identify and validate a robust and reliable molecular prognostic signature and thus improve the accuracy of survival prediction for multiple cohorts of patients with BC.

This study consisted of a training set and 2 validation cohorts, which included 984 patients with BC. The study results indicate that the 8-DRG signature developed herein is significantly associated with poor prognosis in BC and can also properly divide patients into high-risk and low-risk groups in the training and validation sets. The prediction performance of the 8-DGR gene signature proved to be better than that of any single gene in this model. In addition, this 8-DRG signature was still an independent prognostic factor in the multivariate Cox proportional hazards regression analyses. Overall, from the perspective of clinical implications, our 8-DRG prognostic model gives reproducible and reliable results and, thus, can more accurately predict OS of patients with BC.

The 8 DRGs that we identified are *MDC1*, *RPA3*, *MED17*, *DDB2*, *SFPQ*, *XRCC4*, *CYP19A1*, and *PARP3*. In recent years, research has been conducted on some of these genes at the mechanistic level. The *MDC1* expression level is lower in patients with BC. Studies have found that *MDC1* upregulation might suppress the progression of BC by enhancing ERα-mediated transactivation functions, which are associated with the decreased invasion and migration of BC cells.^30,31^ In addition, some results showed that *PARP3* was associated with mediating DNA strand break repair and promoting a transforming growth factor β–induced epithelial-to-mesenchymal transition in patients with BC.^32,33,34^ A recent study showed that silencing *XRCC4* was associated with increased radiosensitivity of triple-negative BC cells.^35^ Acquired *CYP19A1* amplification is an early, specific mechanism of aromatase inhibitor resistance in ERα metastatic BC.^36^ Insulin-like growth factor-binding protein 3 interacts with *SFPQ* in *PARP*-dependent DNA damage repair in triple-negative BC.^37^ *DDB2* is involved in nucleotide excision repair and in other biological processes in normal cells, including transcription and cell cycle regulation.^38^ *DDB2* overexpression was associated with a decrease of adhesion abilities on the glass and plastic areas of BC cells.^39^ Not much evidence has been accumulated on *MED17* and *RPA3* from basic BC research; the successes in studies conducted on these 2 genes during the past decades have been for several other cancers. One such study showed that loss of *MED17* expression in prostate cancer cells was associated with a significant decrease in cellular proliferation, inhibited cell cycle progression, and increased apoptosis.^40^ Moreover, gain-of-function p53 complexes with 2 transcription factors on the promoter (*MED17* and a histone acetyl transferase) was associated with enhanced gene expression to signal cell proliferation and oncogenesis in lung cancer cells.^41^ *RPA3* was found to be associated with hepatocellular carcinoma tumorigenesis, poor patient survival,^10^ poor prognosis in nasopharyngeal cancer, and resistance to radiotherapy.^11^ An elevated *RPA3* expression level is associated with gastric cancer tumorigenesis and poor survival.^12^ However, the specific mechanisms of *MED17* and *RPA3* action in BC need further investigation. We integrated the 8 DRGs into a panel and established a novel multigene signature for predicting the prognosis in BC that showed a strong predictive ability and acted as an independent prognostic molecular factor for patients with BC.

As is well known, BC tissues are divided clinically into different subtypes according to ER, PR, and ERBB2 expression levels.^42,43^ Although the usefulness of ER, PR, and ERBB2 for clinical classification focuses on the selection of responses to treatments, such a conventional examination could not predict prognosis with sufficient accuracy on the basis of the multivariate analyses.^44,45^ Estrogen receptor, PR, and ERBB2 were not identified as independent predictive factors after the multivariate Cox proportional hazards regression analysis. By contrast, the 8-DRG molecular signature was an independent prognostic and predictive factor of BC.

### Limitations

Our preliminary study has some limitations. Considering that the 8-DRG signature that we identified using the TCGA database was verified only in the data sets obtained from the Gene Expression Omnibus database, large-scale multicenter cohorts are needed for external validation. Moreover, precise and rigorous basic experiments must be conducted to further confirm the bioinformatic results obtained here.

Our research identified 8 DRGs associated with the prognosis in BC. The 8-DRG signature showed satisfactory performance in predicting survival in both the training and validation cohorts. Nevertheless, further validations in diverse cohorts are warranted. Moreover, other potential clinical characteristics or new biomarkers might be considered or adjusted to improve the prediction accuracy through majorization of our nomogram model. Precise and rigorous basic experiments are needed to further affirm the bioinformatic results, encouraging us to continue to investigate this project in the future.

## Conclusions

In this study, a novel 8-DRG signature (*MDC1*, *RPA3*, *MED17*, *DDB2*, *SFPQ*, *XRCC4*, *CYP19A1*, and *PARP3*) was successfully identified to predict the survival of patients with BC in both the training and test cohorts. Moreover, the 8-DRG signature is an independent risk factor associated with BC. We hope that it can be applied in clinical treatments or research studies as a potential prognostic biomarker of BC.

## Notes

Supplement. **eTable 1.** Checklist of Items for Reporting a Study Developing or Validating a Multivariable Prediction Model **eTable 2.** Clinic Pathological Characteristics of Extracted Patients With Breast Cancer **eTable 3.** DNA Repair-Related Genes **eTable 4.** Differently Expressed Genes **eTable 5.** The 33 DNA Repair-Related Genes Identified Through the Univariate Cox Analysis **eTable 6.** KEGG Pathways Enriched in High-Risk and Low-Risk Groups by Using GSEA **eFigure 1.** Heatmap of Differentially Expressed Genes Between BC Tissues and Normal Controls **eFigure 2.** Eight DRGs Were Selected to Construct Prediction Model by Multivariate Cox Regression Analysis **eFigure 3.** ROC Curves and Kaplan-Meier Plots of Overall Survival in External Validation Cohorts **eFigure 4.** The Calibration Plots for Predicting Patient 3-Year (A) and 5-Year (B) Overall Survival **eFigure 5.** The functional Enrichment Analysis Included GO Pathway (A) and Biological Process (B) **eFigure 6.** The Functional Enrichment Analysis Included KEGG Pathway (A) and Biological Process (B)
