# Supplementary material for: Prediction of Overall Survival Among Female Patients With Breast Cancer Using a Prognostic Signature Based on 8 DNA Repair–Related Genes
Source: JAMA Netw Open. 2020 Oct 5;3(10):e2014622. doi: 10.1001/jamanetworkopen.2020.14622 (PMC7536586; doi:10.1001/jamanetworkopen.2020.14622)
Supplement: Supplement. — eTable 1. Checklist of Items for Reporting a Study Developing or Validating a Multivariable Prediction Model eTable 2. Clinic Pathological Characteristics of Extracted Patients With Breast Cancer eTable 3. DNA Repair-Related Genes eTable 4. Differently Expressed Genes eTable 5. The 33 DNA Repair-Related Genes Identified Through the Univariate Cox Analysis eTable 6. KEGG Pathways Enriched in High-Risk and Low-Risk Groups by Using GSEA eFigure 1. Heatmap of Differentially Expressed Genes Between BC Tissues and Normal Controls eFigure 2. Eight DRGs Were Selected to Construct Prediction Model by Multivariate Cox Regression Analysis eFigure 3. ROC Curves and Kaplan-Meier Plots of Overall Survival in External Validation Cohorts eFigure 4. The Calibration Plots for Predicting Patient 3-Year (A) and 5-Year (B) Overall Survival eFigure 5. The functional Enrichment Analysis Included GO Pathway (A) and Biological Process (B) eFigure 6. The Functional Enrichment Analysis Included KEGG Pathway (A) and Biological Process (B) [file jamanetwopen-e2014622-s001.pdf]

Supplementary Online Content

Zhang D, Yang S, Li Y, et al. Prediction of overall survival among female patients with breast cancer using a prognostic signature based on 8 DNA repair–related genes. *JAMA Netw Open.* 2020;3(10):e2014622.
doi:10.1001/jamanetworkopen.2020.14622

eTable 1. Checklist of Items for Reporting a Study Developing or Validating a Multivariable Prediction Model
eTable 2. Clinic Pathological Characteristics of Extracted Patients With Breast Cancer
eTable 3. DNA Repair-Related Genes
eTable 4. Differently Expressed Genes
eTable 5. The 33 DNA Repair-Related Genes Identified Through the Univariate Cox Analysis
eTable 6. KEGG Pathways Enriched in High-Risk and Low-Risk Groups by Using GSEA
eFigure 1. Heatmap of Differentially Expressed Genes Between BC Tissues and Normal Controls
eFigure 2. Eight DRGs Were Selected to Construct Prediction Model by Multivariate Cox Regression Analysis
eFigure 3. ROC Curves and Kaplan-Meier Plots of Overall Survival in External Validation Cohorts
eFigure 4. The Calibration Plots for Predicting Patient 3-Year (A) and 5-Year (B) Overall Survival
eFigure 5. The functional Enrichment Analysis Included GO Pathway (A) and Biological Process (B)
eFigure 6. The Functional Enrichment Analysis Included KEGG Pathway (A) and Biological Process (B)

This supplementary material has been provided by the authors to give readers additional information about their work.

© 2020 Zhang D et al. *JAMA Network Open.*

eTable 1. Checklist of Items for Reporting a Study Developing or Validating a Multivariable Prediction Model

		Section/Topic	Item	Development or Validation?	Checklist item	Page
		**Title and abstract**				
		Title	1	D;V	Identify the study as developing and/or validating a multivariable prediction model, the target population, and the outcome to be predicted.	Page 1
		Abstract	2	D;V	Provide a summary of objectives, study design, setting, participants, sample size, predictors, outcome, statistical analysis, results, and conclusions.	Page 5-6
		**Introduction**				
		Background and objectives	3a	D;V	Explain the medical context (including whether diagnostic or prognostic) and rationale for developing or validating the multivariable prediction model, including references to existing models.	Page 7-9
			3b	D;V	Specify the objectives, including whether the study describes the development or validation of the model, or both.	Page 8
		**Methods**				
		Source of data	4a	D;V	Describe the study design or source of data (e.g., randomised trial, cohort, or registry data), separately for the development and validation data sets, if applicable.	Page 10
			4b	D;V	Specify the key study dates, including start of accrual; end of accrual; and, if applicable, end of follow-up.	Page 10, 13
		Participants	5a	D;V	Specify key elements of the study setting (e.g., primary care, secondary care, general population) including number and location of centres.	Page 10, 13
			5b	D;V	Describe eligibility criteria for participants.	Page 10
			5c	D;V	Give details of treatments received, if relevant.	-
		Outcome	6a	D;V	Clearly define the outcome that is predicted by the prediction model, including how and when assessed.	Page 11
			6b	D;V	Report any actions to blind assessment of the outcome to be predicted.	-
		Predictors	7a	D;V	Clearly define all predictors used in developing the multivariable prediction model, including how and when they were measured.	Page 11-12
			7b	D;V	Report any actions to blind assessment of predictors for the outcome and other predictors.	-
		Sample size	8	D;V	Explain how the study size was arrived at.	Page 10
		Missing data	9	D;V	Describe how missing data were handled (e.g., complete-case analysis, single imputation, multiple imputation) with details of any imputation method.	-
		Statistical analysis methods	10a	D	Describe how predictors were handled in the analyses.	Page 10-12
			10b	D	Specify type of model, all model-building procedures (including any predictor selection), and method for internal validation.	Page 11-12
			10c	V	For validation, describe how the predictions were calculated.	Page 11-12
			10d	D;V	Specify all measures used to assess model performance and, if relevant, to compare multiple models.	Page 11-12
			10e	V	Describe any model updating (e.g., recalibration) arising from the validation, if done.	-
		Risk groups	11	D;V	Provide details on how risk groups were created, if done.	-
		Development vs validation	12	V	For validation, identify any differences from the development data in setting, eligibility criteria, outcome, and predictors.	Page 11-12
		**Results**				
		Participants	13a	D;V	Describe the flow of participants through the study, including the number of participants with and without the outcome and, if applicable, a summary of the follow-up time. A diagram may be helpful.	Page 13
			13b	D;V	Describe the characteristics of the participants (basic demographics, clinical features, available predictors), including the number of participants with missing data for predictors and outcome.	Page13

© 2020 Zhang D et al. *JAMA Network Open.*

			13c	V	For validation, show a comparison with the development data of the distribution of important variables (demographics, predictors, and outcome).	Page14
		Model development	14a	D	Specify the number of participants and outcome events in each analysis.	Page13-14
			14b	D	If done, report the unadjusted association between each candidate predictor and outcome.	Page 15
		Model specification	15a	D	Present the full prediction model to allow predictions for individuals (i.e., all regression coefficients, and model intercept or baseline survival at a given time point).	Page 14
			15b	D	Estimates of diagnostic accuracy and their precision (such as 95% confidence intervals)	Page 15
		Model performance	16	D;V	Report performance measures (with CIs) for the prediction model.	Page 14-15
		Model updating	17	V	If done, report the results from any model updating (i.e., model specification, model performance).	-
		**Discussion**				
		Limitations	18	D;V	Discuss any limitations of the study (such as nonrepresentative sample, few events per predictor, missing data).	Page 20-21
		Interpretation	19a	V	For validation, discuss the results with reference to performance in the development data, and any other validation data.	Page 17
			19b	D;V	Give an overall interpretation of the results, considering objectives, limitations, results from similar studies, and other relevant evidence.	Page 17-18
		Implications	20	D;V	Discuss the potential clinical use of the model and implications for future research	Page 18,20,21
		**Other information**				
		Supplementary information	21	D;V	Provide information about the availability of supplementary resources, such as study protocol, Web calculator, and data sets.	Page 10
		Funding	22	D;V	Give the source of funding and the role of the funders for the present study.	Page 22

Note: Items relevant only to the development of a prediction model are denoted by *D*, items relating solely to a validation of a prediction model are denoted by *V*, and items relating to both are denoted *D;V*.

© 2020 Zhang D et al. *JAMA Network Open.*

eTable 2. Clinic Pathological Characteristics of Extracted Patients With Breast Cancer

**Characteristic**		**Group**		**No. of cases (%)**
Age (years)		<60		385(54.46)
		≥60		322(45.54)
Pathological stage		Stage I		123(17.40)
		Stage II		411(58.13)
		Stage III		160(22.63)
		Stage IV		13(1.84)
Pathological T		T1		184(26.03)
		T2		422(59.69)
		T3		76(10.75)
		T4		25(3.54)
Pathological N		N0		344(48.66)
		N1		240(33.95)
		N2		85(12.02)
		N3		38(5.37)
Metastasis		M0		695(98.30)
		M1		12(1.70)
ER		positive		547(77.37)
		negative		160(22.63)
PR		positive		480(67.89)
		negative		227(32.11)
HER2		positive		105(14.85)
		negative		602(85.15)
Vital status		Alive		639(90.38)
		Dead		68(9.62)

Abbreviation: ER, estrogen receptor; PR, progesterone receptor; HER2, human epidermal growth factor 2.

© 2020 Zhang D et al. *JAMA Network Open*.

eTable 3. DNA Repair-Related Genes

Gene Names	
1	AATF
2	ABL1
3	ACTR5
4	AKT1
5	ALKBH1
6	ALKBH2
7	ALKBH3
8	AP5S1
9	AP5Z1
10	APEX1
11	APEX2
12	APITD1
13	APLF
14	APTX
15	ASCC3
16	ASF1A
17	ASTE1
18	ATF2
19	ATM
20	ATMIN
21	ATR
22	ATRIP
23	ATRX
24	ATXN3
25	AXIN2
26	BABAM1
27	BAP1
28	BARD1
29	BAX
30	BAZ1B
31	BCCIP
32	BLM
33	BRAP
34	BRCA1
35	BRCA2
36	BRCC3
37	BRE
38	BRIP1

© 2020 Zhang D et al. *JAMA Network Open*.

39	BTG2
40	BUB1
41	BUB1B
42	C11orf30
43	C17orf70
44	C19orf40
45	CASP3
46	CCNA1
47	CCNA2
48	CCNB1
49	CCND1
50	CCNE1
51	CCNH
52	CCNO
53	CDC14B
54	CDC25A
55	CDC25B
56	CDC25C
57	CDC45
58	CDC6
59	CDH13
60	CDK1
61	CDK2
62	CDK4
63	CDK7
64	CDKN1A
65	CDKN1B
66	CDKN2A
67	CDKN2D
68	CEBPG
69	CEP164
70	CEP170
71	CETN2
72	CHAF1A
73	CHAF1B
74	CHD1L
75	CHD4
76	CHEK1
77	CHEK2
78	CHRNA4

© 2020 Zhang D et al. *JAMA Network Open*.

79	CIB1
80	CINP
81	CLSPN
82	COPS5
83	CRB2
84	CREB1
85	CREBBP
86	CRY1
87	CRY2
88	CSNK1D
89	CSNK1E
90	CUL4A
91	CUL4B
92	CYP19A1
93	CYP1A1
94	DAPK1
95	DBF4
96	DCLRE1A
97	DCLRE1B
98	DCLRE1C
99	DDB1
100	DDB2
101	DDR1
102	DDX1
103	DEK
104	DHX9
105	DMAP1
106	DMC1
107	DNA2
108	DOT1L
109	DTL
110	DTX3L
111	DUSP3
112	DYRK2
113	E2F1
114	E2F2
115	E2F4
116	E2F6
117	EEPD1
118	EGFR

© 2020 Zhang D et al. *JAMA Network Open.*

119	EME1
120	EME2
121	ENDOV
122	EP300
123	EPC2
124	ERBB2
125	ERCC1
126	ERCC2
127	ERCC3
128	ERCC4
129	ERCC5
130	ERCC6
131	ERCC6L2
132	ERCC8
133	ESCO1
134	ESCO2
135	ESR1
136	ETS1
137	EXO1
138	EXO5
139	EYA1
140	EYA2
141	EYA3
142	EYA4
143	FAM175A
144	FAN1
145	FANCA
146	FANCB
147	FANCC
148	FANCD2
149	FANCE
150	FANCF
151	FANCG
152	FANCI
153	FANCL
154	FANCM
155	FBXO18
156	FBXO6
157	FEN1
158	FGF10

© 2020 Zhang D et al. *JAMA Network Open*.

159	FHIT
160	FIGN
161	FIGNL1
162	FOS
163	FOXM1
164	FTO
165	FZR1
166	GADD45A
167	GADD45G
168	GEN1
169	GPS1
170	GSTP1
171	GTF2H1
172	GTF2H2
173	GTF2H2C
174	GTF2H3
175	GTF2H4
176	GTF2H5
177	H2AFX
178	HDAC1
179	HDAC2
180	HELQ
181	HERC2
182	HIC1
183	HINFP
184	HIST3H2A
185	HMGB1
186	HMGB2
187	HUS1
188	HUS1B
189	HUWE1
190	IFI16
191	IGF1
192	IGHMBP2
193	IKBKG
194	INIP
195	INO80
196	INO80D
197	INO80E
198	INTS3

© 2020 Zhang D et al. *JAMA Network Open*.

199	IRS1
200	JMY
201	JUN
202	KAT5
203	KDM2A
204	KIAA0101
205	KIAA0430
206	KIAA2022
207	KIF22
208	KIN
209	KPNA2
210	LIG1
211	LIG3
212	LIG4
213	MAD2L2
214	MBD4
215	MC1R
216	MCM9
217	MCPH1
218	MDC1
219	MDM2
220	MDM4
221	MED17
222	MEIOB
223	MEN1
224	MGME1
225	MGMT
226	MLH1
227	MLH3
228	MMS19
229	MMS22L
230	MNAT1
231	MORF4L1
232	MORF4L2
233	MPG
234	MRE11A
235	MSH2
236	MSH3
237	MSH4
238	MSH5

© 2020 Zhang D et al. *JAMA Network Open*.

239	MSH6
240	MTA1
241	MUM1
242	MUS81
243	MUTYH
244	MYC
245	NABP1
246	NABP2
247	NBN
248	NCOA6
249	NEIL1
250	NEIL2
251	NEIL3
252	NEK1
253	NEK11
254	NFKB1
255	NHEJ1
256	NINL
257	NME1
258	NONO
259	NSMCE1
260	NSMCE2
261	NTHL1
262	NUDT1
263	OGG1
264	OTUB1
265	PALB2
266	PAPD7
267	PARG
268	PARP1
269	PARP2
270	PARP3
271	PARP4
272	PARP9
273	PARPBP
274	PCNA
275	PLK1
276	PLK3
277	PMS1
278	PMS2

© 2020 Zhang D et al. *JAMA Network Open.*

279	PNKP
280	POLA1
281	POLB
282	POLD1
283	POLD2
284	POLD3
285	POLD4
286	POLDIP3
287	POLE
288	POLE2
289	POLE3
290	POLE4
291	POLG
292	POLG2
293	POLH
294	POLI
295	POLK
296	POLL
297	POLM
298	POLN
299	POLQ
300	POLR2A
301	POLR2B
302	POLR2C
303	POLR2D
304	POLR2E
305	POLR2F
306	POLR2G
307	POLR2H
308	POLR2I
309	POLR2J
310	POLR2K
311	POLR2L
312	PPM1D
313	PPP1CA
314	PPP2R2A
315	PPP2R5A
316	PPP2R5B
317	PPP2R5C
318	PPP2R5D

© 2020 Zhang D et al. *JAMA Network Open*.

319	PPP2R5E
320	PPP4C
321	PPP4R2
322	PRKDC
323	PRMT6
324	PRPF19
325	PSMD3
326	PTTG1
327	RAD1
328	RAD17
329	RAD18
330	RAD21
331	RAD23A
332	RAD23B
333	RAD50
334	RAD51
335	RAD51AP1
336	RAD51B
337	RAD51C
338	RAD51D
339	RAD52
340	RAD54B
341	RAD54L
342	RAD9A
343	RAD9B
344	RASSF1
345	RB1
346	RBBP4
347	RBBP7
348	RBBP8
349	RBM14
350	RBX1
351	RDM1
352	REC8
353	RECQL
354	RECQL4
355	RECQL5
356	RELA
357	REV1
358	REV3L

© 2020 Zhang D et al. *JAMA Network Open*.

359	RFC1
360	RFC2
361	RFC3
362	RFC4
363	RFC5
364	RFWD2
365	RFWD3
366	RHNO1
367	RNASEH2A
368	RNF168
369	RNF169
370	RNF8
371	RPA1
372	RPA2
373	RPA3
374	RPA4
375	RPAIN
376	RPS27A
377	RPS27L
378	RPS3
379	RRM2B
380	RTEL1
381	RUVBL1
382	RUVBL2
383	SETD2
384	SETMAR
385	SETX
386	SFPQ
387	SFR1
388	SHFM1
389	SHPRH
390	SIRT1
391	SIRT6
392	SLC30A9
393	SLX1A
394	SLX4
395	SMAD2
396	SMAD3
397	SMAD4
398	SMAD7

© 2020 Zhang D et al. *JAMA Network Open.*

399	SMARCA1
400	SMARCA2
401	SMARCA4
402	SMARCA5
403	SMARCAD1
404	SMARCB1
405	SMARCC2
406	SMARCD1
407	SMARCD2
408	SMC1A
409	SMC2
410	SMC3
411	SMC4
412	SMC5
413	SMC6
414	SMG1
415	SMUG1
416	SMURF2
417	SOD1
418	SP1
419	SPATA22
420	SPIDR
421	SPO11
422	SPP1
423	SPRTN
424	SSRP1
425	STAT1
426	STRA13
427	SUMO1
428	SUPT16H
429	SWI5
430	SWSAP1
431	SYCP1
432	TAOK1
433	TAOK2
434	TAOK3
435	TCEA1
436	TDG
437	TDP1
438	TDP2

© 2020 Zhang D et al. *JAMA Network Open*.

439	TELO2
440	TERF1
441	TERF2
442	TERF2IP
443	TEX12
444	TEX15
445	TICRR
446	TMEM161A
447	TNP1
448	TONSL
449	TOP1
450	TOP2A
451	TOP3A
452	TOPBP1
453	TP53
454	TP53BP1
455	TP73
456	TREX1
457	TREX2
458	TRIP12
459	TRIP13
460	TTC5
461	TWIST1
462	TYMS
463	UBA1
464	UBA52
465	UBB
466	UBC
467	UBE2A
468	UBE2B
469	UBE2D3
470	UBE2I
471	UBE2N
472	UBE2NL
473	UBE2T
474	UBE2U
475	UBE2V2
476	UBE4B
477	UHRF1
478	UIMC1

© 2020 Zhang D et al. *JAMA Network Open*.

479	UNG
480	UPF1
481	USP1
482	USP28
483	USP3
484	USP47
485	USP7
486	UVRAG
487	UVSSA
488	VCP
489	WDR16
490	WDR33
491	WDR48
492	WEE1
493	WHSC1
494	WRN
495	WRNIP1
496	WWP1
497	WWP2
498	XAB2
499	XPA
500	XPC
501	XRCC1
502	XRCC2
503	XRCC3
504	XRCC4
505	XRCC5
506	XRCC6
507	XRCC6BP1
508	YY1
509	ZBTB32
510	ZFYVE26
511	ZNF350
512	ZRANB3
513	ZSWIM7

© 2020 Zhang D et al. *JAMA Network Open.*

eTable 4. Differently Expressed Genes

Gene	P-Value
UBE2T	2.44E-63
CCNB1	2.16E-61
CDK1	5.06E-61
UHRF1	7.83E-61
PTTG1	1.20E-60
PLK1	2.23E-60
CDC25C	4.88E-60
CDC14B	9.82E-60
BUB1	3.72E-59
DTL	4.75E-59
FOXM1	1.66E-58
RNASEH2A	6.14E-58
NEIL3	6.67E-58
EGFR	8.50E-58
EXO1	2.38E-57
KPNA2	3.01E-57
RECQL4	3.73E-57
BUB1B	3.83E-57
RAD51	1.01E-56
TOP2A	3.88E-57
CCNA2	8.36E-56
NME1	1.04E-55
EEPD1	2.25E-55
PPP4C	2.30E-55
PARP1	3.29E-55
EME1	3.51E-55
TRIP13	2.79E-54
E2F1	8.16E-54
FANCI	1.13E-53
RAD54L	1.33E-53
ESCO2	2.18E-53
CDC6	6.12E-53
FEN1	6.51E-53
KIF22	1.16E-52

© 2020 Zhang D et al. *JAMA Network Open.*

CRY2	1.73E-52
CDC45	1.91E-52
RDM1	1.97E-52
RAD51AP1	2.99E-50
H2AFX	2.77E-52
PCNA	4.62E-52
CLSPN	7.41E-51
POLQ	1.32E-50
PPP1CA	5.34E-50
POLR2H	1.14E-49
CDH13	2.10E-49
RFC2	4.53E-49
TONSL	5.01E-49
IGF1	2.87E-48
NABP2	5.55E-48
POLE2	1.39E-47
E2F2	2.87E-47
TYMS	1.04E-46
DNA2	6.86E-45
BLM	1.57E-44
FOS	2.55E-44
RUVBL1	5.33E-44
CDC25A	7.20E-41
BAX	2.64E-43
PARPBP	3.49E-43
BRIP1	6.28E-42
FIGN	2.70E-41
CCNE1	4.31E-41
MORF4L2	5.83E-41
CHEK1	8.04E-41
XRCC2	1.53E-40
FANCD2	1.67E-40
RFC4	3.83E-40
UBA1	6.49E-40
FANCA	1.47E-39
APEX2	1.53E-39

© 2020 Zhang D et al. *JAMA Network Open*.

SMARCA4	3.27E-39
DBF4	4.71E-39
FTO	2.07E-38
CIB1	2.77E-38
PSMD3	2.82E-38
RAD54B	1.73E-37
FBXO6	2.32E-37
RPA3	3.61E-38
RHNO1	3.63E-38
CHAF1B	4.02E-38
SMARCA2	5.64E-38
CHAF1A	6.18E-38
JUN	1.24E-37
CASP3	1.41E-37
SMC4	1.68E-37
REV3L	1.12E-36
RBBP7	3.12E-36
RUVBL2	3.53E-36
CDKN2D	1.06E-35
TICRR	1.42E-35
MEN1	1.91E-35
FANCF	4.39E-35
HELQ	4.85E-35
POLR2K	1.26E-34
PRPF19	3.48E-31
POLR2J	1.79E-32
OTUB1	3.96E-34
BRCA2	7.48E-34
FGF10	1.01E-33
CRB2	9.27E-33
GEN1	1.21E-32
BARD1	1.93E-32
CDKN2A	7.13E-32
COPS5	2.72E-31
LIG1	3.41E-31
SIRT6	5.42E-31

© 2020 Zhang D et al. *JAMA Network Open*.

SLX4	1.95E-30
NUDT1	2.37E-30
CETN2	3.15E-30
HMGB2	6.12E-30
POLR2F	9.07E-30
UBE2A	1.10E-29
POLR2G	1.12E-29
SPP1	8.34E-27
PARP9	2.76E-28
MAD2L2	1.77E-29
SMAD4	3.26E-27
GPS1	4.19E-29
NTHL1	7.53E-29
TWIST1	1.24E-28
POLD2	1.38E-28
SPATA22	1.55E-28
UBE2N	3.94E-27
REV1	5.84E-27
POLD4	6.08E-27
FANCG	8.12E-27
VCP	8.66E-27
TELO2	1.05E-26
CDK7	1.12E-26
SUMO1	1.19E-26
DTX3L	1.20E-26
LIG3	1.35E-26
PNKP	6.44E-25
BABAM1	1.72E-25
DUSP3	2.48E-26
ETS1	5.09E-26
APLF	6.01E-26
MPG	6.16E-26
NSMCE2	7.54E-26
AATF	7.66E-26
RBX1	1.18E-25
CHD4	1.78E-25

© 2020 Zhang D et al. *JAMA Network Open*.

MYC	1.94E-25
POLD1	3.19E-25
SWI5	4.51E-25
AKT1	5.04E-25
RAD21	5.81E-25
UBE2I	7.97E-25
ATXN3	9.40E-25
SHPRH	1.01E-24
APTX	1.17E-24
POLE3	7.61E-22
EME2	2.18E-23
TOP1	1.30E-24
SMUG1	1.65E-24
CHEK2	1.89E-24
BCCIP	2.24E-24
POLE	2.71E-24
CDK4	5.31E-24
WRNIP1	9.01E-24
EPC2	1.05E-23
SMC6	6.09E-23
POLB	6.55E-23
XPA	7.94E-23
POLR2I	1.04E-22
BRCA1	1.44E-22
CDK2	1.55E-22
RAD1	5.63E-22
UVRAG	9.61E-22
NONO	4.39E-21
SIRT1	1.43E-19
RFC5	3.32E-19
STAT1	4.66E-21
HIST3H2A	1.01E-20
ERCC6L2	1.46E-20
AXIN2	3.41E-20
HIC1	6.17E-20
IRS1	6.35E-20

© 2020 Zhang D et al. *JAMA Network Open*.

CDC25B	6.48E-20
EYA1	7.53E-20
FANCB	9.07E-20
AP5Z1	4.92E-19
TERF2IP	6.50E-19
JMY	6.52E-19
BRCC3	6.60E-19
SMARCB1	8.37E-19
SMAD3	1.10E-18
DDR1	1.23E-18
EYA2	1.33E-18
RBM14	3.23E-16
UBE4B	8.56E-17
NSMCE1	2.11E-18
TMEM161A	4.51E-18
POLR2D	1.33E-17
MBD4	2.09E-17
GTF2H1	3.98E-17
RPS27A	4.20E-17
POLK	4.50E-17
CYP1A1	5.34E-17
RFC3	6.01E-17
PPM1D	7.77E-17
UBE2V2	1.12E-16
DHX9	1.15E-16
NEK1	1.18E-16
USP7	1.21E-16
XRCC1	1.58E-16
ATMIN	1.89E-16
GADD45G	1.90E-16
SOD1	3.72E-14
FAN1	3.99E-14
POLI	2.17E-16
IFI16	2.42E-16
SETMAR	3.53E-16
CCNH	4.91E-16

© 2020 Zhang D et al. *JAMA Network Open.*

WRN	5.47E-16
HERC2	5.62E-16
ATM	5.68E-16
WDR48	6.64E-16
PALB2	1.28E-15
SMURF2	2.06E-15
SETX	2.15E-15
XRCC4	2.31E-15
HDAC1	2.62E-15
SMAD2	2.64E-15
ERCC2	3.84E-15
XPC	4.30E-15
SWSAP1	4.76E-15
TAOK2	4.37E-12
EXO5	5.03E-12
MSH6	7.80E-15
UNG	1.33E-14
DDB1	1.36E-14
SMARCA1	1.71E-14
RAD9B	5.96E-14
SYCP1	1.55E-13
PLK3	1.83E-13
MCPH1	2.35E-13
DOT1L	2.82E-13
ERBB2	4.06E-13
PRKDC	4.36E-13
ACTR5	4.40E-13
USP47	6.11E-13
RAD51B	7.52E-13
NABP1	8.23E-13
GSTP1	1.03E-12
BAZ1B	1.17E-12
UBB	1.03E-10
INO80E	1.13E-10
EYA4	1.53E-12
BTG2	2.55E-12

© 2020 Zhang D et al. *JAMA Network Open*.

TDG	2.76E-12
FZR1	3.42E-12
MC1R	3.56E-12
TOPBP1	4.28E-12
ZBTB32	7.57E-12
MSH2	8.12E-12
SPRTN	8.42E-12
RBBP4	1.07E-11
WWP2	1.11E-11
NCOA6	1.31E-11
ABL1	1.46E-11
TDP1	1.59E-11
AP5S1	1.78E-11
CSNK1D	2.23E-11
TCEA1	2.43E-11
NHEJ1	3.78E-09
CUL4A	5.04E-09
CREB1	2.83E-11
SFPQ	3.19E-11
SMC1A	4.09E-11
SMC2	4.49E-11
CCND1	5.16E-11
ERCC8	6.69E-11
SETD2	9.19E-11
FANCC	9.67E-11
HDAC2	1.21E-10
ATRX	1.28E-10
RECQL5	1.32E-10
TEX12	1.61E-10
MORF4L1	3.27E-10
ERCC5	3.32E-10
PARP4	3.64E-10
FHIT	3.65E-10
POLM	5.83E-10
UBE2B	1.43E-07
ZSWIM7	1.45E-07

© 2020 Zhang D et al. *JAMA Network Open*.

APEX1	6.18E-10
DDB2	7.18E-10
RAD23A	7.81E-10
PPP2R5D	9.75E-10
XRCC5	1.51E-09
UPF1	1.85E-09
WEE1	2.69E-09
RFC1	2.73E-09
SP1	2.90E-09
DCLRE1A	3.03E-09
LIG4	6.14E-09
INO80D	6.15E-09
SSRP1	6.72E-09
DYRK2	8.25E-09
HUS1	1.04E-08
CCNA1	1.45E-08
PPP2R5C	1.73E-08
TOP3A	3.16E-06
FANCM	3.64E-06
RFWD3	1.99E-08
SMARCD2	2.19E-08
POLN	2.54E-08
CCNO	2.89E-08
ESR1	3.31E-08
ASTE1	3.46E-08
TERF2	4.60E-08
RAD9A	4.92E-08
INO80	5.48E-08
RAD23B	7.07E-08
MUS81	1.11E-07
RNF8	1.26E-07
KIAA1429	1.81E-07
SMARCC2	2.51E-07
SMC5	3.26E-07
NBN	3.37E-07
TREX2	3.47E-07

© 2020 Zhang D et al. *JAMA Network Open.*

INTS3	3.30E-05
ALKBH1	3.97E-05
RAD51C	3.63E-07
FANCE	4.56E-07
IKBKG	4.67E-07
DCLRE1B	5.64E-07
ALKBH3	6.03E-07
UIMC1	7.02E-07
POLR2L	8.84E-07
MSH3	1.07E-06
RAD52	1.07E-06
ZFYVE26	1.35E-06
RBBP8	1.55E-06
ATF2	1.63E-06
SUPT16H	1.73E-06
UBE2U	3.09E-06
MSH4	3.82E-06
TDP2	3.88E-06
PARP3	7.75E-15
TRIP12	0.000984
RPAIN	0.000986
XRCC3	5.74E-06
POLA1	7.72E-06
MLH1	8.48E-06
NEIL1	8.76E-06
WWP1	9.75E-06
PRMT6	1.02E-05
PARG	1.07E-05
YY1	1.08E-05
RB1	1.30E-05
ALKBH2	1.54E-05
TAOK1	1.64E-05
MUM1	1.75E-05
MLH3	1.76E-05
POLDIP3	1.87E-05
GADD45A	2.06E-05

© 2020 Zhang D et al. *JAMA Network Open*.

MTA1	2.78E-05
PPP2R5B	5.66E-05
TRIP12	0.000984
RPAIN	0.000986
FIGNL1	5.95E-05
RPS27L	6.22E-05
KAT5	0.000116
EP300	0.000121
POLH	0.00014
ESCO1	0.000145
MUTYH	0.000148
PMS1	0.000194
POLR2C	0.000227
POLG2	0.000367
XAB2	0.000378
XRCC6	0.000401
PPP2R2A	0.000645
TEX15	0.000797
NFKB1	0.000839
INIP	0.000877
CHD1L	5.09E-06
BAP1	0.006664
RNF169	0.006979
RPA1	0.000982
RASSF1	0.001054
RAD51D	0.001145
UBC	0.001215
MGME1	0.001841
SFR1	0.002006
POLR2B	0.00236
FANCL	0.002583
CDKN1A	0.002825
REC8	0.003023
SMARCAD1	0.003251
PARP2	0.003638
MDM4	0.003711

© 2020 Zhang D et al. *JAMA Network Open*.

CEP164	0.003759
MDC1	0.004173
POLD3	0.017572
POLL	0.004459
ASCC3	0.023129
USP28	0.02509
ZRANB3	0.004499
USP1	0.004654
USP3	0.004926
KIN	0.00614
SLC30A9	0.007009
SPO11	0.009113
RRM2B	0.00933
MDM2	0.00995
PPP2R5A	0.010057
TERF1	0.01014
DMAP1	0.011619
TTC5	0.011923
CDKN1B	0.014246
ERCC1	0.01461
GTF2H2C	0.014807
SLX1A	0.015732
NEIL2	0.000908
ATR	0.016634
ASCC3	0.023129
CREBBP	0.01737
RECQL	0.01768
DMC1	0.020131
MED17	0.021576
GTF2H2	0.025256
CINP	0.025536
ZNF350	0.03308
RPS3	0.034052
MGMT	0.045253
RELA	0.047308
CUL4B	0.048139

© 2020 Zhang D et al. *JAMA Network Open*.

RTEL1	0.053422
PPP2R5E	0.055747
SPIDR	0.057443
GTF2H3	0.058761
HUS1B	0.059685
KDM2A	0.06183
EYA3	0.064783
GTF2H4	0.074855
E2F4	0.084929
BRAP	0.100292
NINL	0.103809
PPP4R2	0.090742
MNAT1	0.09953
CRY1	0.100233
SMAD7	0.104475
E2F6	0.116266
NEK11	0.125951
CHRNA4	0.12628
RAD18	0.130879
POLR2E	0.136254
UBE2D3	0.136404
ENDOV	0.144908
DDX1	0.157958
MSH5	0.165891
MMS19	0.192747
SMARCA5	0.016365
HMGB1	0.204158
RAD17	0.20824
POLR2A	0.210925
TNP1	0.230678
ERCC4	0.305381
TP53	0.31173
DCLRE1C	0.230753
ERCC6	0.23409
ASF1A	0.25447
UBA52	0.286079

© 2020 Zhang D et al. *JAMA Network Open*.

TAOK3	0.321486
MMS22L	0.326228
HUWE1	0.361281
MCM9	0.36654
SMARCD1	0.369566
UVSSA	0.380279
RPA2	0.388516
GTF2H5	0.392282
POLG	0.408692
RAD50	0.434644
DAPK1	0.44593
SMG1	0.455662
DEK	0.468821
CEBPG	0.471618
RPA4	0.6038
WDR33	0.619451
CSNK1E	0.519255
HINFP	0.540957
UBE2NL	0.570879
CYP19A1	0.576418
OGG1	0.598946
CEP170	0.649642
SMC3	0.655177
ERCC3	0.680003
ATRIP	0.704441
RNF168	0.708775
MEIOB	0.734649
TP73	0.821042
IGHMBP2	0.890871
POLE4	0.897168
PMS2	0.931452
TP53BP1	0.966204

© 2020 Zhang D et al. *JAMA Network Open*.

eTable 5. The 33 DNA Repair-Related Genes Identified Through the Univariate Cox Analysis

Gene	P-Value
XRCC4	9.67E-05
MDC1	0.000135312
POLR2B	0.000522843
PARP3	0.000706385
POLR2C	0.001074071
MED17	0.001976036
RAD23B	0.0022427
UBE2A	0.002856219
ERCC5	0.004523048
TAOK1	0.008428587
COPS5	0.010586473
NONO	0.012394348
SFPQ	0.013396279
DDB2	0.015065351
XRCC1	0.016201615
POLR2K	0.01991322
ERCC1	0.021891509
RPA3	0.026646086
RFC3	0.02818644
RAD54B	0.030476098
ENDOV	0.032081442
RRM2B	0.034083024
RAD1	0.035158125
RPS3	0.035306418
TERF1	0.03545381
FBXO6	0.035680787
UBE2V2	0.037494025
NSMCE2	0.038569488
RAD51	0.04017352
CYP19A1	0.044070583
DMAP1	0.044444104
CCNB1	0.047339099
POLL	0.047773039

© 2020 Zhang D et al. *JAMA Network Open*.

eTable 6. KEGG Pathways Enriched in High-Risk and Low-Risk Groups by Using GSEA

ID	Description	NES	p-value	q-value
hsa04740	Olfactory transduction	-1.524581526	0.001628664	0.200998752
hsa05322	Systemic lupus erythematosus	-1.813281148	0.001828154	0.200998752
hsa04610	Complement and coagulation cascades	-1.885382677	0.001872659	0.200998752
hsa04979	Cholesterol metabolism	-1.61108573	0.007889546	0.635108481
hsa05320	Autoimmune thyroid disease	1.554338669	0.012121212	0.780606061
hsa05034	Alcoholism	-1.3792097	0.028169014	1
hsa05020	Prion diseases	-1.476965942	0.041237113	1
hsa05414	Dilated cardiomyopathy (DCM)	1.328855483	0.045751634	1

Abbreviations: GSEA, gene set enrichment analysis; NES, normalized enrichment score

© 2020 Zhang D et al. *JAMA Network Open*.

eFigure 1. Heatmap of Differentially Expressed Genes Between BC Tissues and Normal Controls

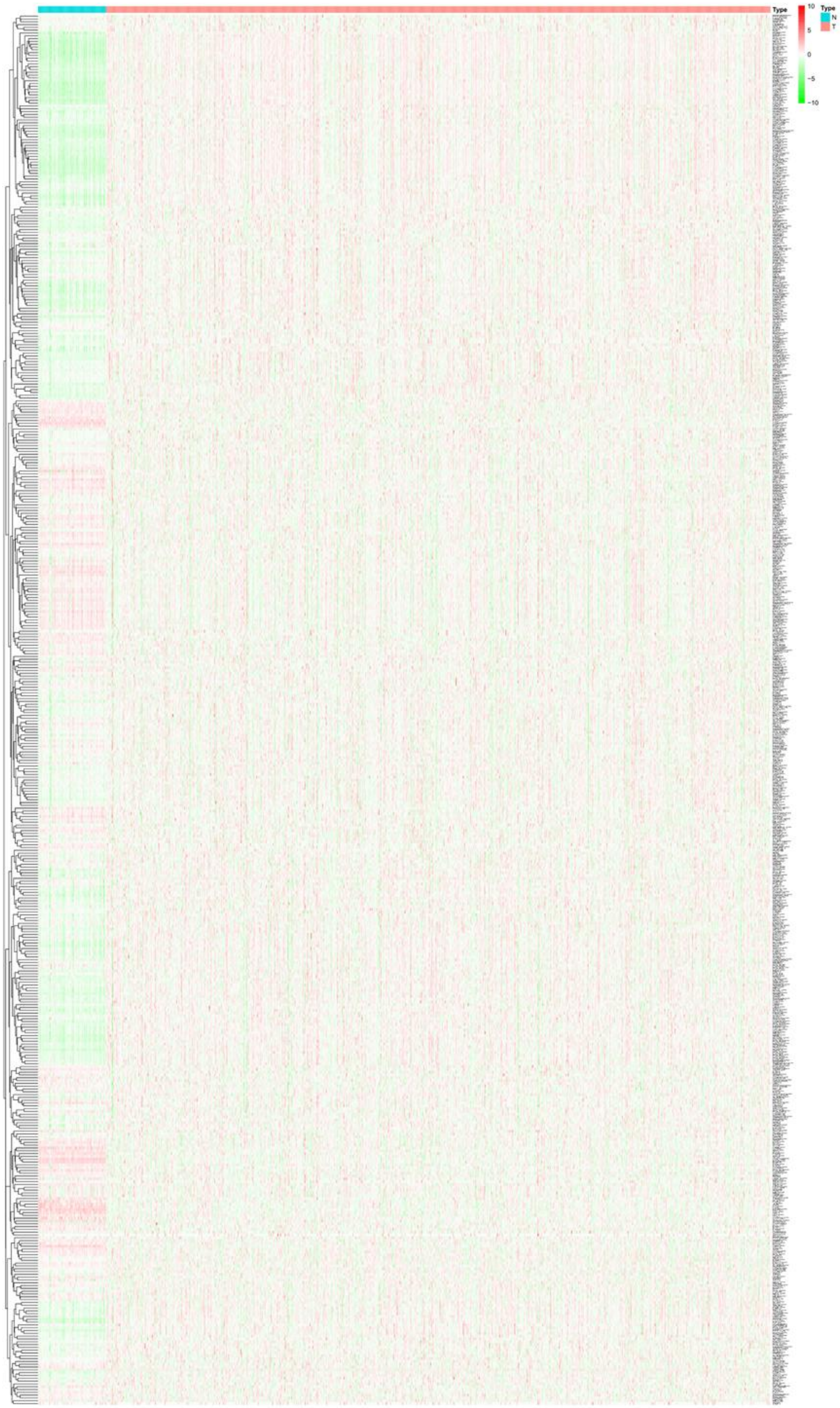

© 2020 Zhang D et al. *JAMA Network Open*.

eFigure 2. Eight DRGs Were Selected to Construct Prediction Model by Multivariate Cox Regression Analysis

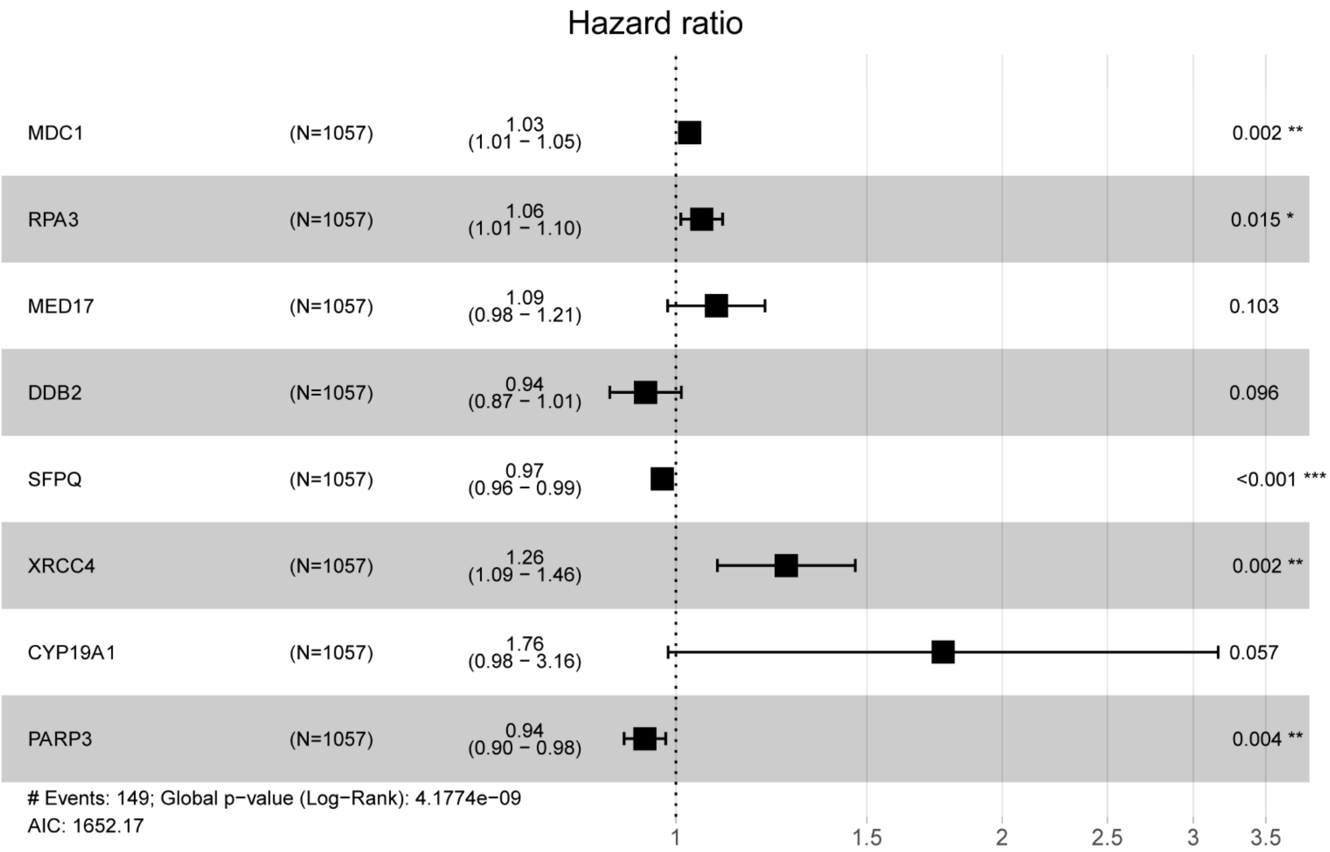

Abbreviation: DRGs, DNA repair-related genes.

© 2020 Zhang D et al. *JAMA Network Open*.

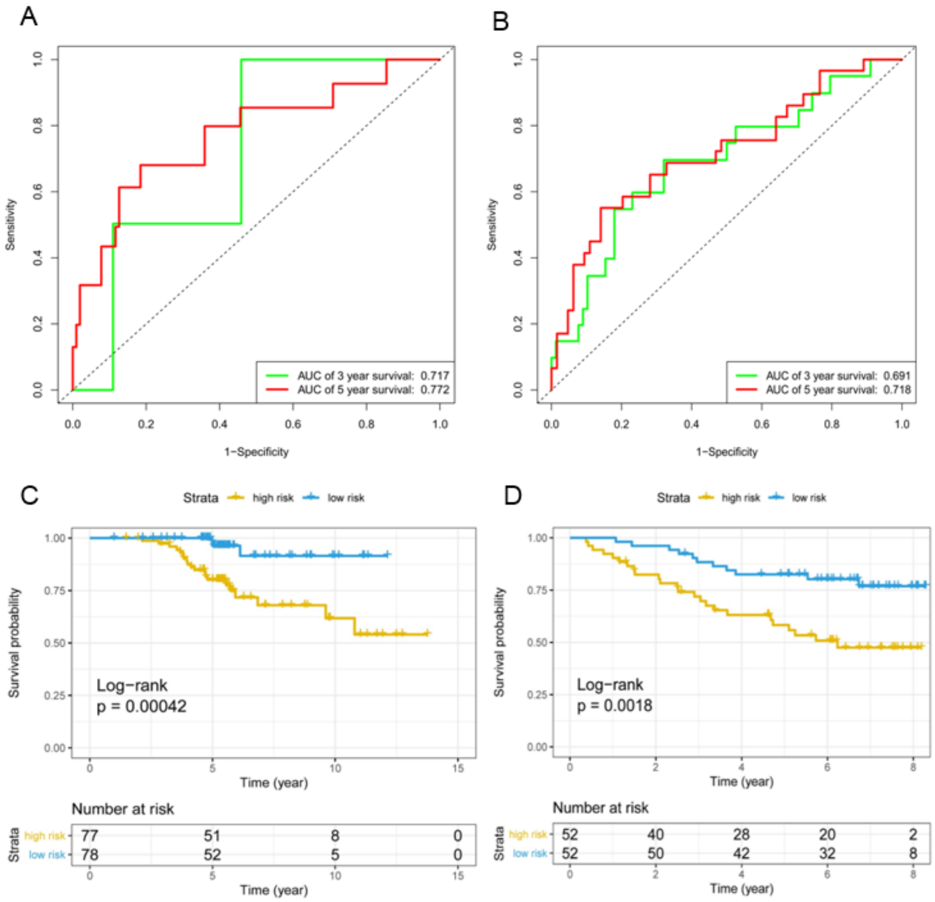

eFigure 3. ROC Curves and Kaplan-Meier Plots of Overall Survival in External Validation Cohorts

A: ROC curves based on the DRG risk score for 3- and 5-year overall survival probability in GSE9893 dataset. B: ROC curves in GSE42568 dataset. C: Kaplan-Meier plots of overall survival according to the expression of eight DRGs in GSE9893 dataset. D: Kaplan-Meier plots of overall survival in GSE42568 dataset.

Abbreviation: DRGs, DNA repair-related genes.

© 2020 Zhang D et al. *JAMA Network Open*.

eFigure 4. The Calibration Plots for Predicting Patient 3-Year (A) and 5-Year (B) Overall Survival

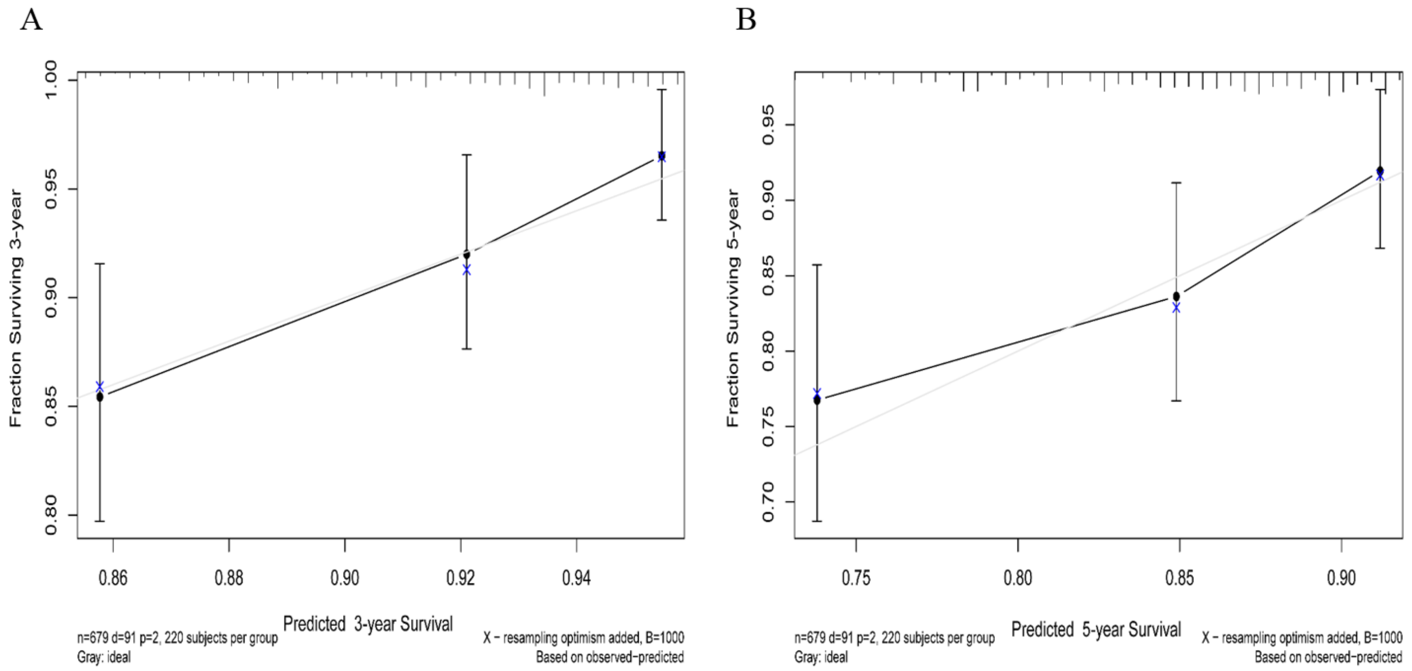

© 2020 Zhang D et al. *JAMA Network Open*.

eFigure 5. The functional Enrichment Analysis Included GO Pathway (A) and Biological Process (B)

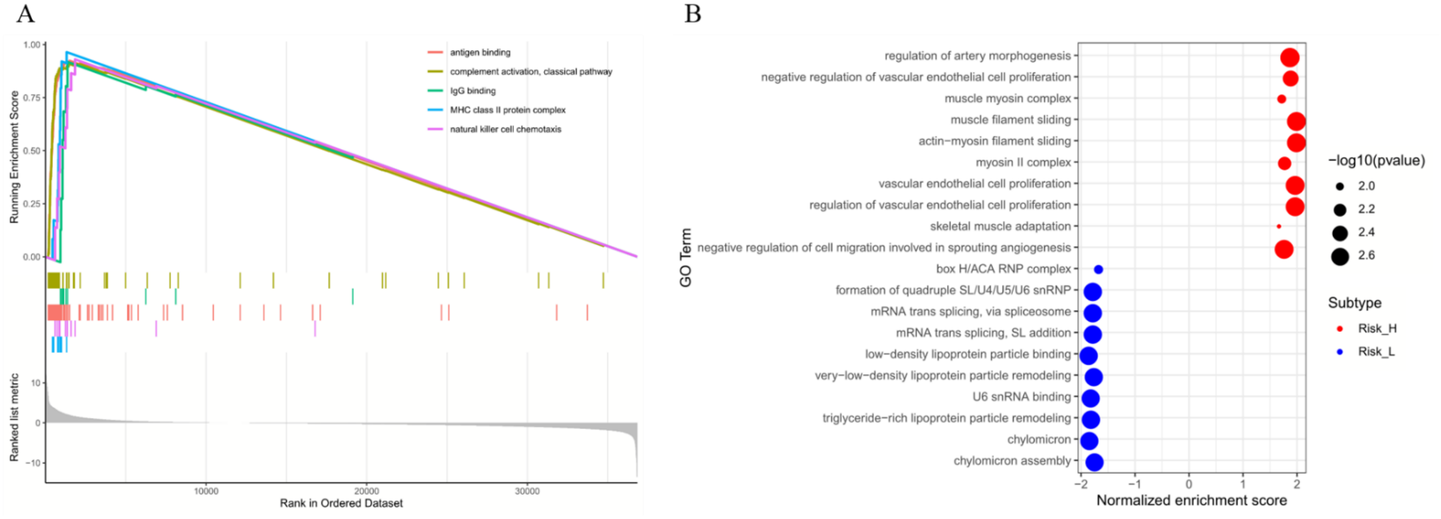

© 2020 Zhang D et al. *JAMA Network Open*.

eFigure 6. The Functional Enrichment Analysis Included KEGG Pathway (A) and Biological Process (B)

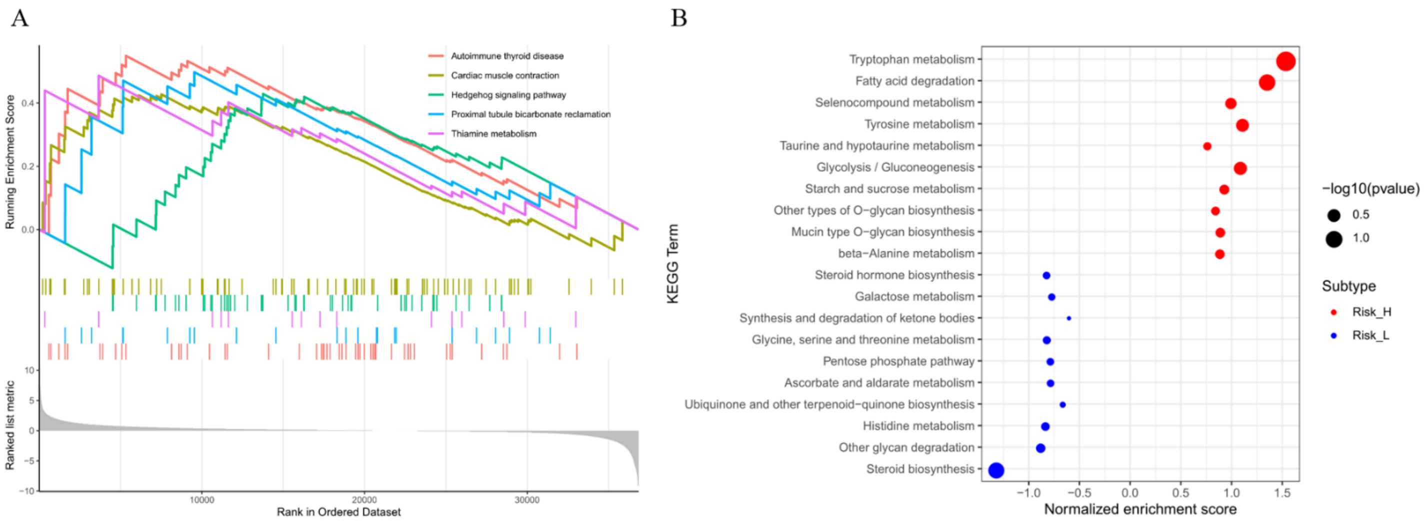

© 2020 Zhang D et al. *JAMA Network Open*.
